# Molecular Dynamics Simulations of Different Nanoparticles at Substrates

**DOI:** 10.3390/ijms25084550

**Published:** 2024-04-21

**Authors:** Małgorzata Borówko, Tomasz Staszewski

**Affiliations:** Department of Theoretical Chemistry, Institute of Chemical Sciences, Faculty of Chemistry, Maria Curie-Skłodowska University in Lublin, 20-031 Lublin, Poland; staszewski@umcs.pl

**Keywords:** molecular dynamics, shapes of nanoparticles, adsorption, self-assembly, pollutant removing

## Abstract

We report the results of large-scale molecular dynamics simulations of adsorption nanoparticles on solid surfaces. The particles were modeled as stiff aggregates of spherical segments. Three types of particles were studied: rods, rectangles, and triangles built of the same number of segments. We show how the particle shape affects the adsorption, the structure of the surface layer, and the degree of the removal of particles from the solvent. The systems with different segment–segment and segment–surface interactions and different concentrations of particles were investigated. The ordered structures formed in adsorption monolayers were also analyzed. The results are consistent with experimental observations.

## 1. Introduction

For over two decades, nanoparticles have found substantial and growing roles in science and technology. We can now produce nanoparticles of various shapes, both homogeneous and heterogeneous, surface-modified particles, including patchy and hairy particles, and many more [1,2,3,4]. The great variety of nanoparticles creates many possibilities for their technological applications in numerous fields, such as heterogeneous catalysis [5], the production of different electronic devices [6], biological and chemical sensors [7], and medicine for the imaging, diagnosis, and treatment of diseases [8].

Nanoparticles not only have many practical applications but are also very interesting from a purely cognitive point of view. Therefore, different systems containing nanoparticles have been studied, mainly various bulk phases and fluid–fluid interfaces [9,10]. Most of the research has concentrated on the self-assembly of nanoparticles leading to the formation of new ordered structures. Several excellent reviews have been made available in summarizing the self-assembly strategies according to different shapes and interactions [11,12].

Relatively little attention has been paid to the study of nanoparticles on solid surfaces. However, the experimental and theoretical investigations have shown that the structure of the adsorption layer containing nanoparticles can be controlled by the surface potential [13,14,15,16,17,18,19,20,21,22,23,24,25,26]. Various theoretical methods have been used for modeling the interaction of nanoparticles with solid surfaces, including computer simulations [15,16,17,19,20,21,22,26] and density functional theory [18,23,24].

The behavior of different particles near solid surfaces has been studied. In particular, studies of hairy particles have focused on the changes in the internal structure of the polymer coating on the different substrates, and the surface-induced self-assembly [16,17,18,19,20,21]. Moreover, the influence of the substrate on the behavior of spherical patchy particles [22] and, above all, Janus particles have been investigated [23,24,25]. Much effort has also been devoted to the study of pore-confined Janus particles [25,26] to explore how interactions between particles, the nature of pore walls, and their separation influence self-assembly.

The process of adsorption of nanoparticles on solid surfaces has rarely been investigated. Usually, nanoparticles are considered as the adsorbent, but not adsorbate [27,28]. However, recently the importance of the adsorption of nanoparticles on solids has been increasing. Many commercial products containing nanoparticles release them into the environment. They should, therefore, be treated as a new type of potentially toxic pollutant. For example, silver particles are a very popular aseptic agent, but they can accumulate in the food chain and pose a threat to the environment. Research is ongoing on methods for removing nanoparticles from the environment. One of the most promising methods is just adsorption on solid surfaces [27].

Research on the adsorption of nanoparticles on solids was stimulated by achievements in understanding the adsorption mechanism of small molecules, polymers, and peptides. Nevertheless, this issue has not been the subject of systematic theoretical research and a number of detailed questions have not yet found a satisfactory answer. So far, the influence of particle shape on the amount of adsorption and the morphology of surface layers has not been investigated. Our research aimed to fill this gap. The influence of shape on the behavior of nanoparticles at the liquid–liquid interface has already been discussed in detail [9]. Moreover, the shape effect in cellular uptake of nanoparticles has been confirmed [29]. The results of these studies inspired us to investigate the influence of the shape of nanoparticles on the adsorption on the surfaces of solids.

In this work, we analyzed the behavior of rods, rectangular plates, and triangular plates on different surfaces. We dealt with nanoplates because such nanoparticles can have particularly interesting catalytic [30], optical [31], and antibacterial [32] properties.

We focused on the role of particle shape in the adsorption and assembly. Our goals were twofold. Firstly, we wanted to answer the question of how the particle’s shape influences the amount of adsorbed particles and, thus, the ability to remove them from the solvent. Secondly, we studied the structure of surface layers in different conditions. For this purpose, we changed the interactions between particles, their interactions with the substrate, and the system density. Finally, we concentrated on the morphology of monolayer films formed on the substrate via adsorption.

Our simulations proved that the particle shape can play a key role in the adsorption, removal of nanoparticles from the bulk phase, and surface-induced assembly. However, quantitative effects associated with the particle shape considerably depend on the interactions in the system and the concentration of nanoparticles. We have formulated a few detailed conclusions regarding the behavior of nanoparticles in different systems. In general, the behavior of rigid rods on solid surfaces is different than that of plates. For example, we found that in good adsorption conditions, there are more rods than plates and more triangles than rectangles in the surface layer. Moreover, surface layers built of plates considerably differ from those formed by the rods, they are thicker and more “rough”. Rods are adsorbed parallel to the surface, in contrast, the plates are slightly tilted relative to the substrate. In dense monolayers, rods form ordered structures more easily than plates. The shape of the particle determines the type of ordered phases formed.

This paper is organized as follows. In Section 2, we present the results concerning the adsorption of different nanoparticles, their removal efficiency, the structure of surface layers, and the morphology of adsorbed monolayers. Section 3 describes the model used and the simulation protocol. Section 4 concludes the study.

## 2. Results and Discussion

### 2.1. Description of Studied Systems

We address here the issue of the behavior of nanoparticles of various shapes on solid surfaces. It is well-known that the nanoparticle shape influences its adsorption properties. Therefore, it is desirable to investigate how the shape of nanoparticles affects the behavior on solid surfaces. The scope of the study was quite wide. We investigated the effects of nanoparticle shape, the adsorption energy, and the inter-particle interactions on the adsorption process and the structure of the surface layer. We varied the system parameters to achieve results that would reveal general trends in the behavior relevant to practical applications. We considered the adsorption of two model particles, inert and attractive ones, on the weak ($\varepsilon_{s}^{*}=2.0$), moderately strong ($\varepsilon_{s}^{*}=3.5$) and strong surface ($\varepsilon_{s}^{*}=5.0$). The average density of the systems varies from $\rho_{0}=0.02$ to $\rho_{0}=0.06$.

### 2.2. Adsorption of Nanoparticles on Solid Surfaces

To characterize an amount of adsorption, we have computed the following quantities: (i) the excess adsorption, (ii) the real adsorption, and (iii) the degree of the removal of the particles from the bulk phase. The calculations were made for different sets of system parameters.

**Figure 1:**
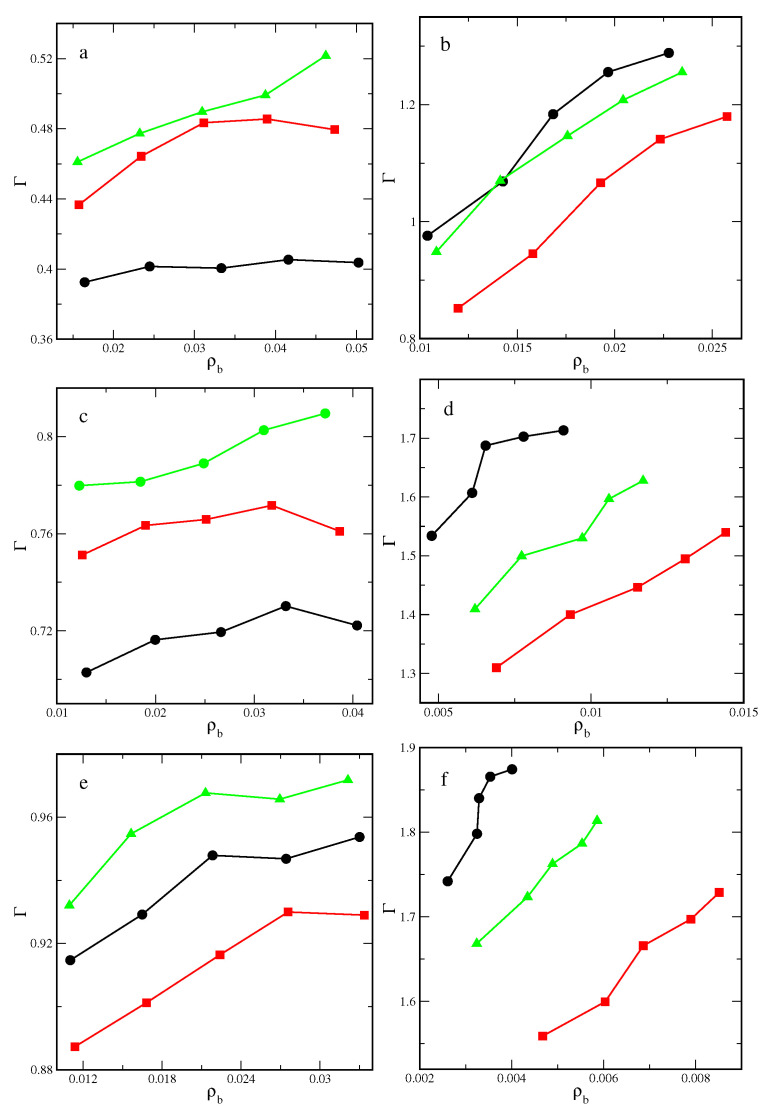
Excess adsorption isotherms of inert (**a**,**c**,**e**) and attractive (**b**,**d**,**f**) rods (black circles), rectangles (red squares), and triangles (green triangles) on the surfaces with $\varepsilon_{s}^{*}=$ 2.0 (**a**,**b**), 3.5 (**c**,**d**) and 5.0 (**e**,**f**). Symbols correspond to simulation points. Lines serve as a guide to the eye.

We begin with the discussion of excess adsorption isotherms presented in Figure 1. The results for inert and attractive particles are shown in the left and right panels, respectively. We see here that the excess adsorption is always greater for stronger surfaces and for attractive particles. The strongest influence of the inter-particle attraction is observed for rods that have the surface most exposed to contact with other particles.

The most important finding is that particle shape significantly affects excess adsorption. However, this influence depends on the system parameters. We observe the following relationships:

- Γ(rods)<Γ(rectangles)<Γ(triangles), for inert particles and weaker surfaces;
- Γ(rectangles)<Γ(rods)<Γ(triangles), for inert particles and the strongest adsorbent;
- Γ(rectangles)<Γ(triangles)<Γ(rods), for attractive particles.

Notice that for inert particles and weak surfaces, the adsorption of plates is significantly stronger than the adsorption of rods, while the opposite effect is observed for attractive particles. The attractive plates are strongly sucked out from the surface layer by particles in the bulk phase. Moreover, the excess adsorption of rectangles is always lower than that of triangles.

We counted the real adsorption from Equation (6) using the thickness (*h*) of the surface layer estimated from the density profiles. Determination of the *h* is not straightforward because the density gradually tends to the bulk value, and even small fluctuations in density can affect the estimation. We assumed that the dividing surface between the adsorption layer and the bulk phase is located at the distance from the surface at which the local density of segments differs from its bulk value more than 5% [20].

**Figure 2:**
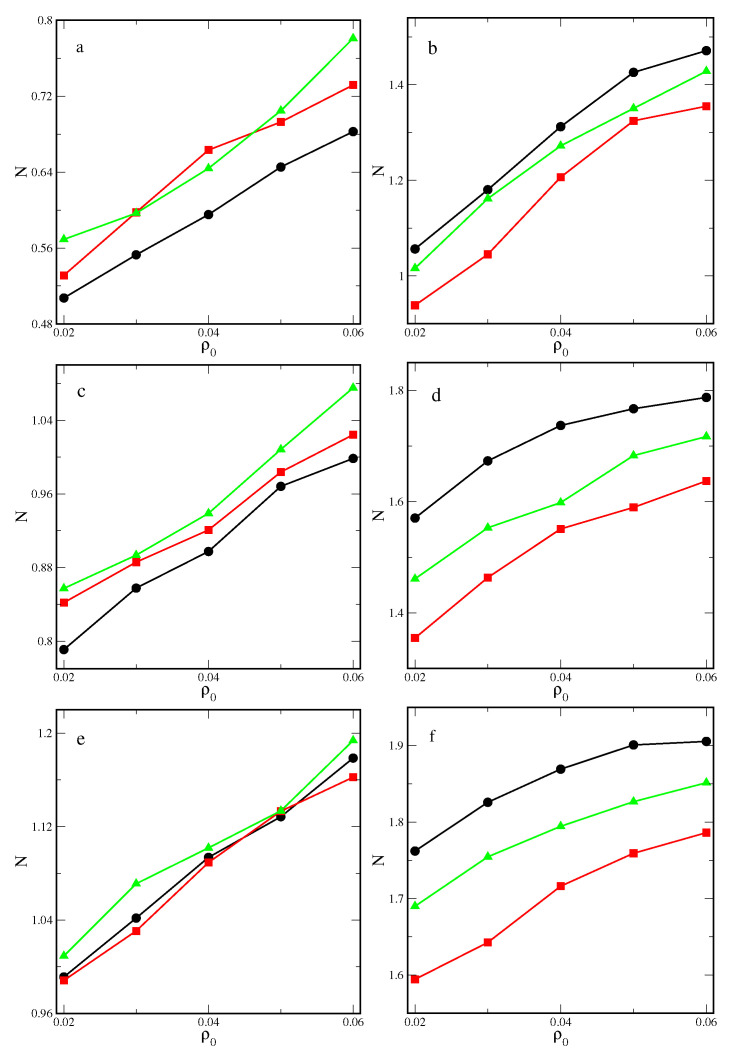
Real adsorption of inert (**a**,**c**,**e**) and attractive (**b**,**d**,**f**) rods (black circles), rectangles (red squares), and triangles (green triangles) on the surfaces with $\varepsilon_{s}^{*}=$ 2.0 (**a**,**b**), 3.5 (**c**,**d**), and 5.0 (**e**,**f**). Symbols correspond to simulation points. Lines serve as a guide to the eye.

Figure 2 shows the real adsorption plotted as the function of the average system density, $\rho_{0}$. The effect of the shape of particles on the real adsorption, *N*, is qualitatively the same as in the case of the excess adsorption, Γ. Surprisingly, for inert particles and the strongest surface, the real adsorption isotherms for different particles are similar. However, for attractive particles, the impact of shape is significant. This is a consequence of the complex interplay between particle–particle and particle–surface interactions in the inhomogeneous system.

**Figure 3:**
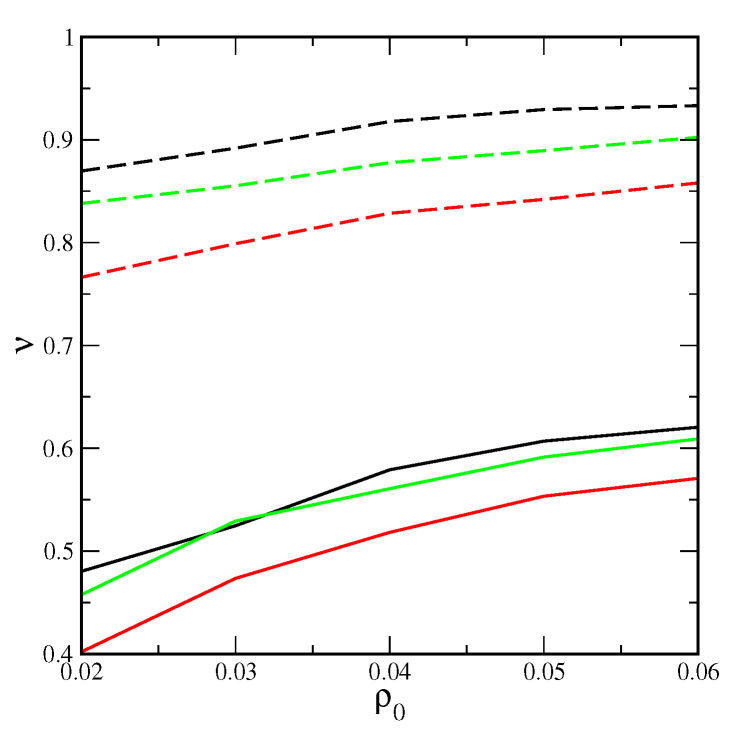
The degree of the removing of the particles from the bulk phase as a function of the average system density for attractive rods (black lines), rectangles (red lines), and triangles (green lines) on the surfaces with $\varepsilon_{s}^{*}\;=\;$ 2.0 (solid lines) and 5.0 (dashed lines).

In addition, we also evaluated the degree of the removal of the particles from the bulk phase. We found that the shape of the particles affects the efficiency of their removal from the solvent. The degree of removal of particles, ν, always rises strongly with increasing adsorption energy and attractive interaction between particles. Figure 3 displays the results for attractive particles. We see here that the degree of removal considerably increases with increasing the average density, $\rho_{0}$. Moreover, for the strongest adsorbent, the removal of rods is the most effective. Completely different results were obtained for inert particles.

In this case, the degree of removal changes only slightly with the $\rho_{0}$. Moreover, the removal efficiency is considerably lower, for example, when $\varepsilon_{s}^{*}=5.0$ and $\rho_{0}=0.06$, ν(rods)=0.45, ν(rectangles)=0.44, and ν(triangles)=0.46. This means that the removal of triangular plates is the easiest, but the shape of the effect is insignificant.

### 2.3. Structure of Surface Layer

To complete the picture of the behavior of nanoparticles on a solid surface, we computed the selected structural characteristics of studied systems and analyzed the relevant snapshots. We discuss here the following functions determined for considered particles: (i) the segment density profiles obtained for different densities and the fixed energy of interaction with the substrate, (ii) the segment density profiles obtained for different energies of interaction with the substrate and the fixed density, and (iii) the orientation-order parameter as a function of the energy of interaction with the substrate.

**Figure 4:**
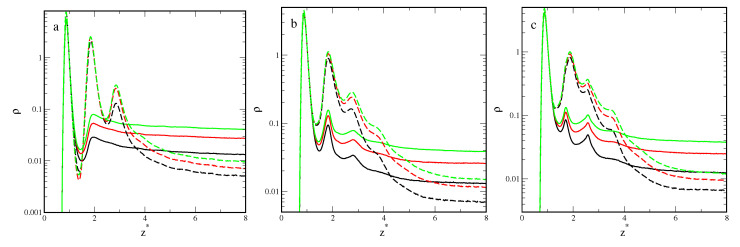
Density profiles of segments for inert (solid lines) and attractive (dashed lines) rods (**a**), rectangles (**b**), and triangles (**c**) for $\varepsilon_{s}^{*}=3.5$ and different densities $\rho_{0}$: 0.02 (black lines), 0.04 (red lines), and 0.06 (green lines). The abscissa is scaled logarithmically.

Figure 4 presents the density profiles of segments for inert (solid lines) and attractive (dashed lines) rods (a), rectangles (b), and triangles (c), plotted for $\varepsilon_{s}^{*}=3.5$ and different densities, $\rho_{0}$: 0.02 (black lines), 0.04 (red lines), and 0.06 (green lines). The increase in the average system density causes an increase in the segment density near the substrate. All density profiles $\rho (z^{*})$ show one well-pronounced and high maximum at $z^{*}\approx 0.89$. This indicates the presence of a relatively dense adsorption layer on the surface. Then, the density falls to a minimum at $z^{*}\approx 1.53$ or $z^{*}\approx 1.40$ for inert and attractive particles, respectively. It is worth noting that these minima are lower than the bulk density. The more particles there are in the first layer, the deeper the minimum.

**Figure 5:**
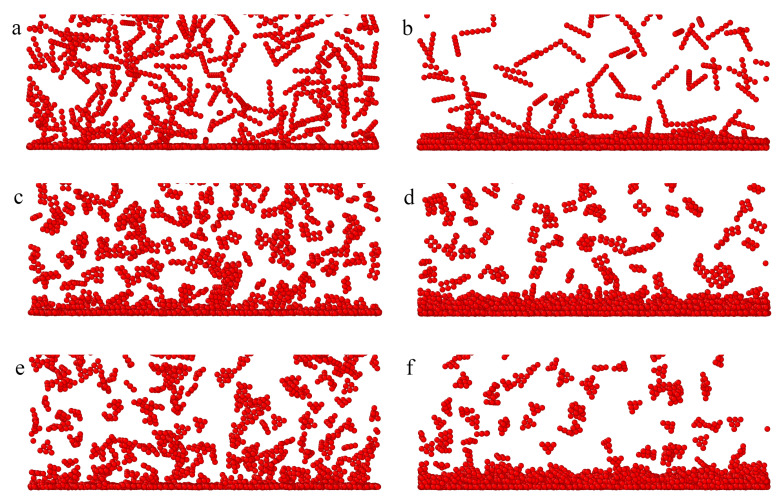
Side views of fragments of the equilibrium configurations of inert (**a**,**c**,**e**) and attractive (**b**,**d**,**f**) rods (**a**,**b**), rectangles (**c**,**d**), and triangles (**e**,**f**) adsorbed on the surface with $\varepsilon_{s}^{*}=3.5$; $\rho_{0}=0.02$.

Density profiles were estimated for different particles that differed significantly, which confirmed disparate morphologies of the adsorbed layers. In the case of inert rods, apart from the high first maximum, the profiles exhibit a small and wide peak indicating the formation of the second layer. This suggests that inert rods are adsorbed parallel to the surface. For attractive rods, however, we see a series of three gradually decreasing peaks in the density profiles. Molecules adsorbed in the first layer attract others and a multilayer surface phase is built (see Figure 4a). This conclusion is confirmed by the snapshots presented in Figure 5, where we see the adsorption monolayer for inert particles (part a) and the multilayer surface phase for attractive particles (part b).

The segment density profiles obtained for the plates are considerably different than those calculated for rods. The surface layer is thicker, the density more slowly decreases to the bulk values. The differences are particularly visible in the case of inert particles. For plates, there are three peaks in the density profile. Likely some of the rectangles and triangles are set at an angle to the surface. Indeed, one can see it in the snapshots presented in Figure 5c,e.

In the case of attractive particles, the outer part of the adsorption layer of plates is more diffuse than that for rods (Figure 4). For both plates, the adsorbed phase is more irregular and “rough” (Figure 5).

**Figure 6:**
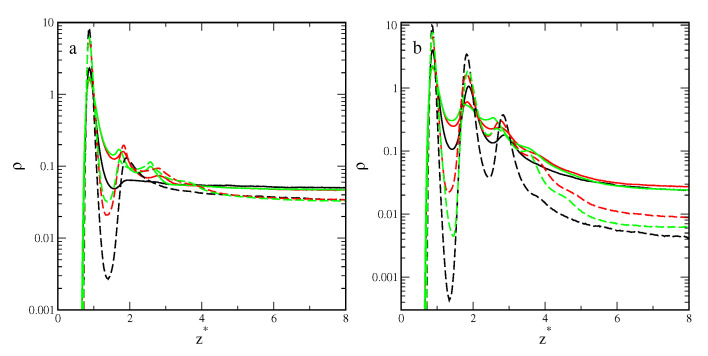
Density profiles of segments for inert (**a**) and attractive (**b**) rods (black lines), rectangles (red lines), and triangles (green lines) for surfaces with $\varepsilon_{s}^{*}=2.0$ (solid lines) and $\varepsilon_{s}^{*}=5.0$ (dashed lines); $\rho_{0}=0.06$. The abscissa is scaled logarithmically.

To further explore the role of particle shape in adsorption, in Figure 6 we plotted density profiles of different particles for $\rho_{0}=0.06$ and two adsorbents—the weakest (solid lines) and the strongest (dashed lines).

Figure 6a shows density profiles, $\rho (z^{*})$, for the inert particles adsorbed on these adsorbents. If $\varepsilon_{s}^{*}=2.0$, all density profiles, $\rho (z^{*})$, show one well-pronounced and high maximum at $z^{*}\approx 0.89$. This indicates the presence of a dense adsorption layer on the surface. For rods, there is a shallow minimum at $z^{*}\approx 1.59$. Then, the density smoothly tends to the bulk value. However, two low peaks are also observed for the plates, indicating that the second and third layers of segments are present. The profiles for rectangles and triangles are very similar. As the adsorption energy increases to $\varepsilon_{s}^{*}=5.0$, the density profiles considerably change. For all particles, there are deep minima at $z^{*}\approx 1.40$. For rods and rectangles, the second peaks are higher and sharper. Only a small third peak is observed for plates. Then, the densities gradually decrease to the bulk density. The surface layer is now much thicker than for the weak substrate.

Also for attractive particles (Figure 6b), the profiles obtained for rods are considerably different than those determined for plates. For $\varepsilon_{s}^{*}=2.0$, in the density profile of rods, we see three sharp peaks, while for plates the density decreases almost gradually and only two minor peaks are observed. As the adsorption energy increases to $\varepsilon_{s}^{*}=5.0$, the second peaks become higher also for plates.

The high segment density at $z^{*}\approx 0.89$ and the lower density in the outer part of the adsorption layer suggest that the particles lie directly on the surface. The first-layer particles occupy a larger surface area and maximize the interactions with the surface.

**Figure 7:**
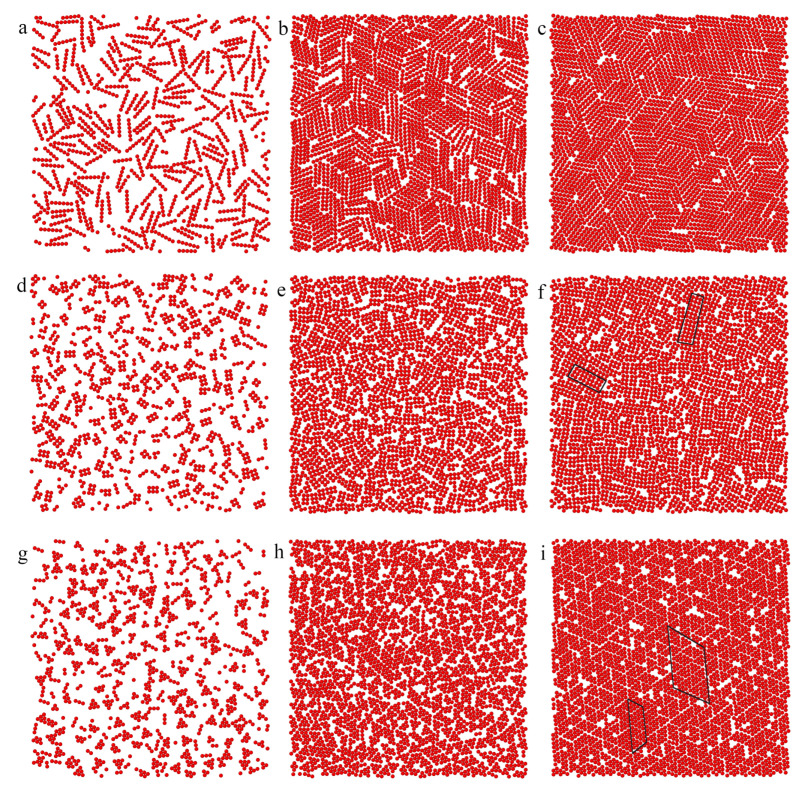
Views from above on the particles with at least one segment in contact with the surface ($z^{*}<1$). The left column (**a**,**d**,**g**) presents results for $\varepsilon_{s}^{*}=2.0$ and inert particles, the middle column (**b**,**e**,**h**) is for $\varepsilon_{s}^{*}=5.0$ and inert particles, while the right column (**c**,**f**,**i**) is for $\varepsilon_{s}^{*}=5.0$ and attractive particles. The layers containing rods, rectangles, and triangles are shown in the top (**a**–**c**), middle (**d**–**f**), and bottom rows (**g**–**i**), respectively. Ordered patches are marked as black quadrilaterals.

To provide a deeper insight into the morphology of the surface layer, we present the particles with at least one segment in contact with the surface ($z^{*}<1$). This corresponds to the arrangement of the particles adsorbed in the first layer. Figure 7 shows the exemplary distributions of particles on the surfaces with $\varepsilon_{s}^{*}=2.0$ and 5. We begin with a discussion of the rod’s behavior (the top row). Most of the rods lie on the substrate. Notice that single segments or their clusters containing fewer than six segments in the snapshots correspond to molecules at an angle to the substrate. Inert rods are randomly distributed on the weak adsorbent (part a). It is interesting, however, that on the strong surface (part b), inert rods assemble into clusters where they are arranged parallel to each other. These clusters are randomly oriented. We see here islands of the smectic phase. Obviously, more particles are adsorbed on the strong surface. The density of the first layer is high, and packing effects are significant. The entropy-driven ordering is observed. In the case of attractive rods, both enthalpy and entropy contributions to the free energy support the formation of the smectic phase (see part c). We discuss this issue in detail in the next paragraph.

In lower rows, we show results for plates. We see here that considerably fewer inert plates lie on the weak substrate (parts d, g). With increasing the energy $\varepsilon_{s}^{*}$ more particles are adsorbed parallel to the surface. In dense phases, inert plates form small islands of ordered structures. This tendency is reinforced for attractive particles (Figure 7f,i). Rectangles are arranged parallel to each other, while triangles form “zippers” built of inversely oriented particles.

We also analyzed the second adsorption layer (not seen here) and found that only rods are parallel to the surface, but the plates are randomly oriented to the substrate. The rods form “low walls” at the surface, while for plates, the outer part of the surface layer is chaotic.

**Figure 8:**
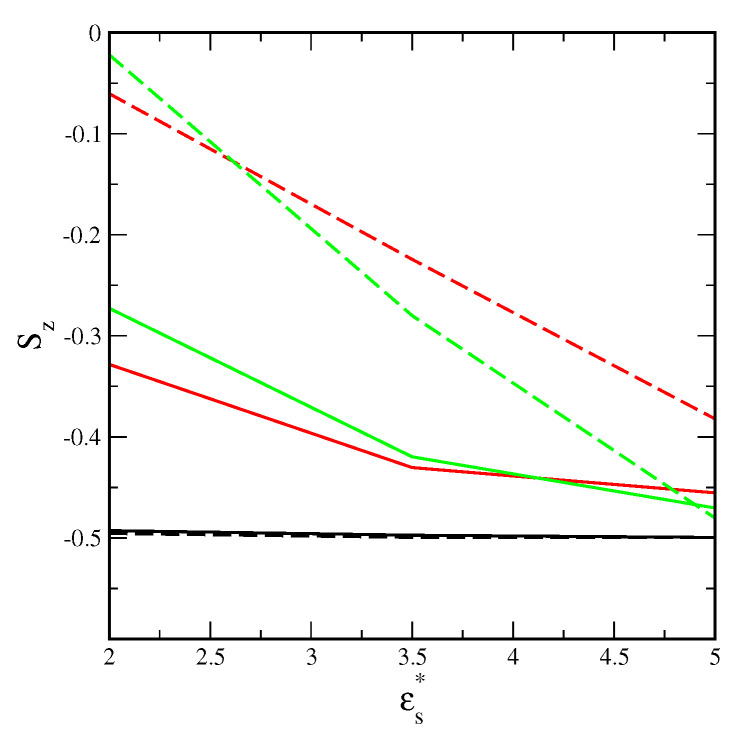
The orientational–order parameter $S_{z}$ as a function of the energy $\varepsilon_{s}^{*}$ for inert (solid lines) and attractive (dashed lines) rods (black lines), rectangles (red lines), and triangles (green lines); $\rho_{0}=0.06$.

To quantify the orientation effects, we calculated the orientation-order parameter $S_{z}$ shown in Figure 8. Indeed, rods always adsorb parallel to the surface, the order parameter $S_{z}=-0.5$, and does not change with increasing adsorption energy. Contrary, a considerable part of plates are tilted to the surface. Nevertheless, the orientation parameter $S_{z}$ still is low and varies from −0.48 to −0.02 (for attractive triangles). This corresponds to the dihedral angle with the surface $7^{\circ }<\left(90^{\circ }-\theta \right)<34^{\circ }$. The parameter $S_{z}$ decreases with the increasing adsorption energy. Thus more plates lie parallel to the stronger adsorbents. For the weakest adsorbent, in the first layer, the parallel adsorption of rectangles is slightly stronger than the parallel adsorption of triangles. The opposite is true for strong adsorbents. A crossing point shifts to lower adsorption energy for attractive plates. Moreover, attractive interactions between particles cause more particles to be set at a certain angle to the surface. It is likely that interactions with particles in the bulk phase cause flat nanoparticles to be unable to align perfectly parallel to the surface.

### 2.4. Surface-Induced Assembly of Nanoparticles

We carried out an additional series of simulations for systems in which the monolayer adsorption occurs. For this purpose, we considered particles with a low average density $\rho_{0}=0.01$ in contact with strongly attractive surfaces with $\varepsilon_{s}^{*}=5.0$ and $\varepsilon_{s}^{*}=6.5$. The analysis of the density profiles always confirmed the formation of only one adsorption layer.

**Figure 9:**
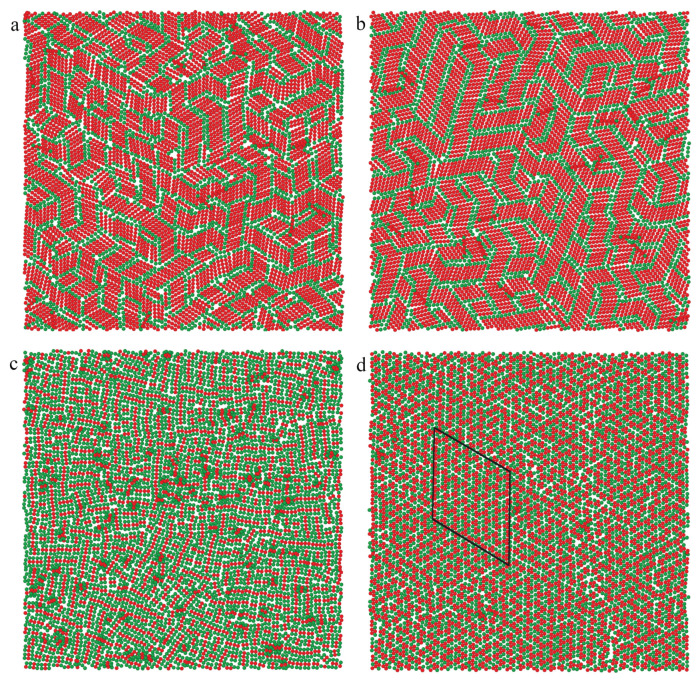
Views from above on the equilibrium configurations of inert (**a**) rods and attractive rods (**b**), rectangles (**c**), and triangles (**d**) adsorbed on the surface with $\varepsilon_{s}^{*}=6.5$; $\rho_{0}=0.01$. The ordered patche is marked as a black quadrilateral.

The structures obtained for both surfaces are very similar. This observation seems to be slightly surprising. However, in considered systems, almost all particles are absorbed even on the weaker adsorbent. Therefore, an increase of $\varepsilon_{s}^{*}$ only slightly affects the amount of adsorption and the structure of the structure of the monolayer. In Figure 9, we show exemplary equilibrium configurations of rods, rectangles, and triangles on the surface with adsorption energy $\varepsilon_{s}^{*}=6.5$. To facilitate the analysis of the structure, rod ends, and vertices of rectangles and triangles are marked in green. We see that the nanoparticles have a tendency to assemble into ordered structures. Rods form patches of the smectic phase where the particles are arranged in parallel. These islands are randomly oriented towards each other. Notice that such an ordering is observed also for inert rods and the attractive interactions between particles only slightly increase the size of the ordered lobes. Significantly different results were obtained for the plates considered. In order not to lengthen the article, we do not show snapshots of monolayers built of inert plates. For the inert rectangles, the particles are randomly distributed on the surface. For the attractive rectangles, however, we see chaotically distributed short chains glued together with the longer sides (see Figure 9c). Under assumed conditions, the rectangles do not form extensive ordered assemblies on the time scale of our simulations. In turn, the inert triangles assemble into small ordered patches, while for attractive triangles, large ordered islands are visible (marked with a black line in Figure 9d). The triangles form alternatively oriented stripes with vertexes located on a triangular lattice. As has been mentioned, due to attractive interactions between particles, their adsorption on the substrate increases. This means that the packing of the monolayer also increases and the tendency to entropy-driven ordering intensifies.

The results of our simulations are qualitatively consistent with those obtained from previous theoretical [33,34,35] and experimental [36,37,38,39] studies devoted to the shape-directed assembly of nanoparticles.

The monolayer adsorption of nanoparticles on the substrate corresponds to covering the surface with tiles. For sufficiently dense monolayers, close-packed structures are obtained. It is well-known that rods tend to assemble into the smectic phase [36]. The plates can form more complex space-filling coverings. The simplest are Platonic surface tilings, which consist of regular triangles, squares, or hexagons. Glotzer and coworkers [33,34,35] studied the self-assembly of hard polygon plates in two-dimensional systems using Monte Carlo simulations. They considered inert plates with excluded volume interactions only and particles with edge-to-edge and edge-to-vertex attractive interactions or both. They found that triangles and squares can assemble without attraction between the nanoplates and due solely to entropy. The vertexes of these plates are located at triangular and square lattices, respectively. However, the behavior of rectangles was different. In the case of rectangles with low values of aspect ratio degeneration, rectangular tilings were observed [34]. On the other hand, rectangles with aspect ratio 2 have a tendency to self-assemble the random domino (parquet) tiling [35] because of their higher entropy.

Unfortunately, there are no experimental results regarding the structure of layers formed on solid surfaces solely as a result of the adsorption of nanoparticles. Methods to assemble nanoparticles in precise two- and three-dimensional architectures are described in the review [39]. The predominant technique simply dries colloidal suspensions of nanoparticles on substrates. However, in the interfacial assembly method, the ordered structure is formed at a liquid–liquid interface and transferred onto glass substrates for solvent evaporation [38].

The structures predicted by our simulations have been found in real systems [36,37,38,39]. For example, Ye et al. [38] synthesized colloidal upconversion nanophosphors of various shapes and compositions and investigated their assembly on glass substrates. They have shown that nanorods form islands of smectic phases (Figure 1d in [38]). After slow evaporation of concentrated $LaF_{3}$ triangular nanoplates from solution in toluene/hexane, a hexagonally close-packed (hcp) superlattice formed on the TEM grid [37]. In this superlattice, the nanoplates lay flat on the face and self-assemble into nanoarrays via edge-to-edge formation (Figure 2a in [37]). The structure of the adsorbed layer shown in Figure 9b is the same.

## 3. Materials and Methods

### 3.1. Model of the Studied Systems

We considered three types of nanoparticles in contact with solid surfaces within the coarse-grained model [40]. The particle consisted of six spheres (segments) connected together to form rods and rectangular and triangular plates. The considered particles were stiff. Each segment had a diameter σ. We used an implicit solvent model [41,42], which treats solvents as a continuous medium surrounding the particles. This meant that no solvent molecules were present in the system, but all interactions should be considered as effective, solvent-mediated ones.

The interactions between the particle’s segments were modeled through the truncated and shifted Lennard–Jones potential [43].

(1) $$u=\left\{\begin{array}{cc} 4\varepsilon_{p}\left[{\left(\sigma /r\right)}^{12}-{\left(\sigma /r\right)}^{6}\;\right]-\Delta u\left(r\right), & \;\;\;r<r_{cut},\\ 0, & \;\;\;\mathrm{otherwise}, \end{array}\right.$$

where

(2) $$\Delta u\left(r\right)=u\left(r_{cut}\right)+\left(r-r_{cut}\right)\partial u\left(r_{cut}\right)/\partial r.$$

In the above, $r_{cut}$ is the cutoff distance for the particle’s segments interactions, while $\varepsilon_{p}$ characterizes the strength of effective interactions between them. To switch on or switch off attractive interactions, we use the cutoff distance. For attractive interactions, $r_{cut}=2.5\sigma$ while, for repulsive interactions, $r_{cut}=\sigma$. By changing the values of the parameters $\varepsilon_{p}$ and $r_{cut}$ we can mimic the presence of various solvents.

The energy of interactions of segments with the solid surfaces is given by the Lennard–Jones (9-3) equation [44,45,46].

(3) $$v_{k}\left(z\right)=\left\{\begin{array}{cc} \frac{2}{15}\varepsilon_{s}\left[{\left(\sigma /z\right)}^{9}-{\left(\sigma /z\right)}^{3}\;\right], & \;\;\;z<z_{cut},\\ 0, & \;\;\;\mathrm{otherwise}, \end{array}\right.$$

where $z_{cut}$ is the cutoff distance, while $\varepsilon_{s}$ is the parameter characterizing interactions of segments with the surface. The potential (3) is commonly used to describe the interactions of particles with a flat structureless solid surface [44,45,46]. As previously, to switch on or switch off attractive interactions with the surface, we used the cutoff distance parameters. The energy of the wall potential was shifted so that it fell to zero at the cutoff distance [15,18].

We introduce the standard units commonly used in molecular simulations [47]. The diameter of spheres is the distance unit, σ, the segment–segment energy parameter for softly repulsive segments, ε is the energy unit, and the mass of a single segment is the mass unity, *m*, and the unit of temperature is $\varepsilon /k_{B}$, where $k_{B}$ is the Boltzmann constant. The basic unit of time is $\tau =\sigma \sqrt{\varepsilon /m}$. We neglect the gravity effects.

As usual, we defined the reduced (dimensionless) quantities [47], such as the reduced distances $l^{*}=l/\sigma$, the reduced energies $E^{*}=E/\varepsilon$, and the reduced temperature $T^{*}=k_{B}T/\varepsilon$.

We also defined the number density of segments $\rho =6N_{p}/V$, where $N_{p}$ denoted the number of particles and *V* was the volume of the system.

In the implicit solvent model, effectively repulsive interactions between particles correspond to solvophilic (hydrophilic) particles. Particle–solvent interactions are more energetically profitable than particle–particle ones. Repulsive segments do not form clusters. In this paper, we call particles built of repulsive segments “inert particles” (inert towards each other). Inversely, attractive effective particle–particle interactions mimic the solvophobic (hydrophobic) particles that have a tendency to aggregate.

We carried out simulations for attractive substrates. In this case, our model can be used to describe distinct real systems, namely: (i) non-polar particles on non-polar (hydrophobic) substrates or (ii) polar particles on polar (hydrophilic) substrates. For example, in the case of metallic nanoparticles, strong attractive interactions with silica substrate are observed while organic particles are attracted by non-polar methylene-capped silicon surface [14].

We used the energy parameters assumed in the previous simulations of nanoparticles near the surfaces [15,16,17,18,19,20,21,48].

### 3.2. Simulation Protocol

We performed molecular dynamics simulations using the LAMMPS package [45,46], followed by post-processing by means of in-house codes to evaluate the system observables. Nose–Hoover thermostat was applied to regulate the temperature. The simulation box was a cuboid of reduced dimensions equal to $L_{x}^{*}$, $L_{y}^{*}$, $L_{z}^{*}$ along the axes *x*, *y*, and *z*, respectively. Standard periodic boundary conditions in the *x* and *y* directions were assumed. The solid surface was located at $z^{*}=0.0$. From the top, the system was closed by a repulsive wall. Temperature was kept at $T^{*}=1$.

All simulations were carried out for 7200 segments (1200 particles). Our simulations were performed for repulsive and attractive particles. We carried out two series of simulations. Firstly, we modeled adsorption on three surfaces characterized by the energy parameters: $\varepsilon_{s}$=2.0, 3.5, and 5.0. For each set of parameters, we studied systems with the average densities of segments, $\rho_{0}$: 0.02, 0.03, 0.04, 0.05, 0.06. We assumed that $L_{x}^{*}=L_{y}^{*}=60\sigma$ or 85σ and the average density of the system was regulated by changing the box height ($L_{z}^{*}$). Secondly, we investigated the monolayers formed on the surfaces with $\varepsilon_{s}^{*}$ = 5 and 6.5. For this purpose, in the system with $\rho_{0}=0.02$ we increased the surface, leaving the height of the box and the number of particles unchanged. Then the average density of segments was $\rho_{0}=0.01$.

Each system was equilibrated using at least $10^{8}$ time steps until its total energy reached a constant level, at which it fluctuated around a mean value. The production runs had at least $10^{7}$ time steps. During the simulation, we calculated the density profiles of segments and total energy.

Examples of the equilibrium configurations were presented using the OVITO [49].

### 3.3. Computed System Observables

The thermodynamic measure of the adsorption is the excess adsorption (per unity of the surface area) defined as

(4) $$\Gamma =\int_{0}^{h}\left(\rho \left(z\right)-\rho_{b}\right)dz,$$

where ρ(z) is the local density of segments and $\rho_{b}$ is the density of the segments in the bulk phase that is determined from the density profile. At equilibrium, far away from the surface, the segment density reaches a plateau corresponding to the bulk density. Obviously, the local density of the particles is $\rho_{p}\left(z\right)=\rho \left(z\right)/6$.

The excess adsorption can be obtained experimentally from the following equation

(5) $$\Gamma =V(\rho_{0}-\rho_{b}),$$

where *V* is the volume of the adsorption system, $\rho_{0}$ and $\rho_{b}$ are densities before and after adsorption.

In turn, the real adsorption (a number of segments in the adsorbed layer per unity of the surface area) is given by

(6) $$N=\int_{0}^{h}\rho \left(z\right)dz,$$

where *h* is the thickness of the surface layer. We estimated h from the segment density profile [20]. For extremely low densities in the bulk phase and strong adsorption, Γ≈N.

We defined the degree of the removal of the particles from the bulk phase as

(7) $$\nu =(1-\rho_{0}/\rho_{b})$$

To characterize the orientation of particles adsorbed at the surface we determined the angle between a rod and the normal to the substrate (ϕ) and the dihedral angle between a plate and the plane normal to the surface (α). We considered the particles with at least one segment touching the surface. Then, the orientation of rods and plates was characterized by the orientational-order parameter

(8) $$S_{z}=3cos^{2}\left(\theta \right)-1$$

where θ=ϕ or θ=α for rods and plates, respectively. The values of $S_{z}$ vary between −0.5 (for particles parallel to the substrate) and 1 (for particles perpendicular to the substrate).

## 4. Conclusions

In this work, we reported the results of large-scale molecular dynamics simulations of adsorption nanoparticles on solid surfaces. The influence of the shape of particles on their behavior on the substrates was discussed. We considered three types of particles, rods, rectangles, and triangles built of the same number of segments.

Firstly, we analyzed the adsorption of the particles on the solid surfaces. For this purpose, excess adsorption isotherms, real adsorption isotherms, and the degree of the removal of the particles from the bulk phase were evaluated for all studied systems. We found the following:

- The shape of particles considerably affects the adsorption and the degree of the removal of the particles from the bulk phase;
- In good adsorption conditions, there are more rods than plates and more triangles than rectangles in the surface layer;
- For attractive particles, the degree of the removal of the particles considerably depends on the shape, and it is easier to remove rods from the environment than plates, while for inert particles, the shape effect is insignificant.

Secondly, we investigated the impact of the shape of nanoparticles on the structure of adsorbed layers and showed the following:

- For inert particles, monolayer adsorption occurs, while for attractive ones, the multilayer surface phase is formed;
- Surface layers built of plates considerably differ from those formed by the rods, they are thicker and more “rough”;
- Rods are adsorbed parallel to the surface, while the plates are slightly tilted relative to the substrate;
- All inert particles are randomly distributed over the surface of weak adsorbents, while on the strongest surface, plates form small ordered aggregates and rods assemble into larger patches;
- Attractive particles assemble into ordered islands in which rods form patches of smectic phase, rectangles lie parallel to each other, while triangles form “zippers” built of inversely oriented particles.

Finally, we studied dense adsorption monolayers formed at very strong adsorbents. In such monolayers, we found the following:

- Inert and attractive rods form quite large patches of smectic phase;
- Inert rectangles do not form any ordered structures, while attractive rectangles are glued together with the longer sides into short strings;
- Inert triangles assemble into small ordered patches, while attractive triangles form large islands of alternatively oriented stripes with vertexes located on a triangular lattice.

These results are qualitatively consistent with those obtained from previous theoretical [33,34,35] and experimental [36,37,38,39] studies of the assembly of nanoparticles. Our research shows that adsorption on solid surfaces can be used to produce ordered two-dimensional structures of nanoparticles.

In summary, we have shown how the shape of nanoparticles influences their adsorption on solid surfaces, the structure of the surface layer, and the possibility of the formation of two-dimensional ordered structures on the substrate. We hope that our results will be a starting point for further theoretical and experimental research in the field of nanotechnology and environmental protection.

## Data Availability

The original contributions presented in the study are included in the articl, further inquiries can be directed to the corresponding author.

## References

[1] Su H., Hurd Price C.A., Jing L., Tian Q., Liu J., Qian K. (2019). Janus particles: Design, preparation, and biomedical applications. Mater. Today Bio.

[2] Duan Y., Zhao X., Sun M., Hao H. (2021). Research Advances in the Synthesis, Application, Assembly, and Calculation of Janus Materials. Ind. Eng. Chem. Res..

[3] Moffitt M.G. (2013). Self-Assembly of Polymer Brush-Functionalized Inorganic Nanoparticles: From Hairy Balls to Smart Molecular Mimics. J. Phys. Chem. Lett..

[4] Pengo P., Şologan M., Pasquato L., Guida F., Pacor S., Tossi A., Stellacci F., Marson D., Boccardo S., Pricl S. (2017). Gold nanoparticles with patterned surface monolayers for nanomedicine: Current perspectives. Eur. Biophys. J..

[5] Bell A.T. (2003). The Impact of Nanoscience on Heterogeneous Catalysis. Science.

[6] Lu W., Lieber C. (2007). Nanoelectronics From the Bottom Up. Nat. Mater..

[7] Wu X., Hao C., Kumar J., Kuang H., Kotov N.A., Liz-Marzán L.M., Xu C. (2018). Environmentally responsive plasmonic nanoassemblies for biosensing. Chem. Soc. Rev..

[8] Langer R., Weissleder R. (2015). Nanotechnology. JAMA.

[9] Borówko M., Staszewski T. (2023). Hybrid Nanoparticles at Fluid–Fluid Interfaces: Insight from Theory and Simulation. Int. J. Mol. Sci..

[10] Shi S., Russell T.P. (2018). Nanoparticle Assembly at Liquid–Liquid Interfaces: From the Nanoscale to Mesoscale. Adv. Mater..

[11] Thorkelsson K., Bai P., Xu T. (2015). Self-assembly and applications of anisotropic nanomaterials: A review. Nano Today.

[12] Xu W., Li Z., Yin Y. (2018). Colloidal Assembly Approaches to Micro/Nanostructures of Complex Morphologies. Small.

[13] Che J., Jawaid A., Grabowski C.A., Yi Y.J., Louis G.C., Ramakrishnan S., Vaia R.A. (2016). Stability of Polymer Grafted Nanoparticle Monolayers: Impact of Architecture and Polymer-Substrate Interactions on Dewetting. ACS Macro Lett..

[14] Che J., Park K., Grabowski C.A., Jawaid A., Kelley J., Koerner H., Vaia R.A. (2016). Preparation of Ordered Monolayers of Polymer Grafted Nanoparticles: Impact of Architecture, Concentration, and Substrate Surface Energy. Macromolecules.

[15] Ethier J.G., Hall L.M. (2018). Modeling individual and pairs of adsorbed polymer-grafted nanoparticles: Structure and entanglements. Soft Matter.

[16] Ethier J.G., Hall L.M. (2018). Structure and Entanglement Network of Model Polymer-Grafted Nanoparticle Monolayers. Macromolecules.

[17] Ventura Rosales I.E., Rovigatti L., Bianchi E., Likos C.N., Locatelli E. (2020). Shape control of soft patchy nanoparticles under confinement. Nanoscale.

[18] Borówko M., Sokołowski S., Staszewski T., Pizio O. (2018). Adsorption of hairy particles with mobile ligands: Molecular dynamics and density functional study. J. Chem. Phys..

[19] Staszewski T., Borówko M. (2018). Molecular dynamics simulations of mono-tethered particles at solid surfaces. Phys. Chem. Chem. Phys..

[20] Staszewski T., Borówko M., Boguta P. (2022). Adsorption of Polymer-Tethered Particles on Solid Surfaces. J. Phys. Chem. B.

[21] Borówko M., Staszewski T. (2022). Shape Transformations and Self-Assembly of Hairy Particles under Confinement. Int. J. Mol. Sci..

[22] Dias C.S., Araújo N.A.M., Telo da Gama M.M. (2013). Non-equilibrium adsorption of 2AnB patchy colloids on substrates. Soft Matter.

[23] Rosenthal G., Klapp S.H.L. (2011). Ordering of amphiphilic Janus particles at planar walls: A density functional study. J. Chem. Phys..

[24] Rosenthal G., Klapp S.H.L. (2012). Micelle and Bilayer Formation of Amphiphilic Janus Particles in a Slit-Pore. Int. J. Mol. Sci..

[25] Kobayashi Y., Arai N. (2016). Self-assembly of Janus nanoparticles with a hydrophobic hemisphere in nanotubes. Soft Matter.

[26] Baran L., Borówko M., Rżysko W. (2020). Self-Assembly of Amphiphilic Janus Particles Confined between Two Solid Surfaces. J. Phys. Chem. C.

[27] Syafiuddin A., Salmiati S., Hadibarata T., Kueh A.B.H., Salim M.R., Zaini M.A.A. (2018). Silver Nanoparticles in the Water Environment in Malaysia: Inspection, characterization, removal, modeling, and future perspective. Sci. Rep..

[28] Borówko M., Staszewski T. (2021). Adsorption on Ligand-Tethered Nanoparticles. Int. J. Mol. Sci..

[29] Li Y., Kröger M., Liu W.K. (2015). Shape effect in cellular uptake of PEGylated nanoparticles: Comparison between sphere, rod, cube and disk. Nanoscale.

[30] Kavan L., Yum J.H., Grätzel M. (2011). Graphene Nanoplatelets Outperforming Platinum as the Electrocatalyst in Co-Bipyridine-Mediated Dye-Sensitized Solar Cells. Nano Lett..

[31] Lu L., Kobayashi A., Tawa K., Ozaki Y. (2006). Silver Nanoplates with Special Shapes: Controlled Synthesis and Their Surface Plasmon Resonance and Surface-Enhanced Raman Scattering Properties. Chem. Mater..

[32] Pal S., Tak Y.K., Song J.M. (2007). Does the Antibacterial Activity of Silver Nanoparticles Depend on the Shape of the Nanoparticle? A Study of the Gram-Negative Bacterium *Escherichia coli*. Appl. Environ. Microbiol..

[33] Millan J.A., Ortiz D., van Anders G., Glotzer S.C. (2014). Self-Assembly of Archimedean Tilings with Enthalpically and Entropically Patchy Polygons. ACS Nano.

[34] Millan J.A., Ortiz D., Glotzer S.C. (2015). Effect of shape on the self-assembly of faceted patchy nanoplates with irregular shape into tiling patterns. Soft Matter.

[35] Harper E.S., Marson R.L., Anderson J.A., van Anders G., Glotzer S.C. (2015). Shape allophiles improve entropic assembly. Soft Matter.

[36] Dennis F., Gardner J.S.E., Smalyukh I.I. (2011). Towards Reconfigurable Optical Metamaterials: Colloidal Nanoparticle Self-Assembly and Self-Alignment in Liquid Crystals. Mol. Cryst. Liq. Cryst..

[37] Zhang Y.W., Sun X., Si R., You L.P., Yan C.H. (2005). Single-Crystalline and Monodisperse LaF3 Triangular Nanoplates from a Single-Source Precursor. J. Am. Chem. Soc..

[38] Ye X., Collins J., Kang Y., Chen J., Chen D., Yodh A., Murray C. (2010). Morphologically controlled synthesis of colloidal upconversion nanophosphors and their shape-directed self-assembly. Proc. Natl. Acad. Sci. USA.

[39] Chen P.Z., Pollit L., Jones L., Gu F.X. (2018). Functional Two- and Three-Dimensional Architectures of Immobilized Metal Nanoparticles. Chem.

[40] Kmiecik S., Gront D., Kolinski M., Wieteska L., Dawid A.E., Kolinski A. (2016). Coarse-Grained Protein Models and Their Applications. Chem. Rev..

[41] Leach A. (2001). Molecular Modeling: Principles and Applications.

[42] Roux B., Simonson T. (1999). Implicit solvent models. Biophys. Chem..

[43] Toxvaerd S., Dyre J.C. (2011). Communication: Shifted forces in molecular dynamics. J. Chem. Phys..

[44] Abraham F.F., Singh Y. (1977). The structure of a hard-sphere fluid in contact with a soft repulsive wall. J. Chem. Phys..

[45] Plimpton S. (1995). Fast Parallel Algorithms for Short-Range Molecular Dynamics. J. Comp. Phys..

[46] Thompson A.P., Aktulga H.M., Berger R., Bolintineanu D.S., Brown W.M., Crozier P.S., in’ t Veld P.J., Kohlmeyer A., Moore S.G., Nguyen T.D. (2022). LAMMPS—A flexible simulation tool for particle-based materials modeling at the atomic, meso, and continuum scales. Comp. Phys. Comm..

[47] Frenkel D., Smit B. (1996). Understanding Molecular Simulation: From Algorithms to Applications.

[48] Lafitte T., Kumar S.K., Panagiotopoulos A.Z. (2014). Self-assembly of polymer-grafted nanoparticles in thin films. Soft Matter.

[49] Stukowski A. (2010). Visualization and analysis of atomistic simulation data with OVITO-the Open Visualization Tool. Model. Simul. Mater. Sci. Eng..

